# Supplementary material for: A doxycycline- and light-inducible Cre recombinase mouse model for optogenetic genome editing
Source: Nat Commun. 2022 Oct 28;13:6442. doi: 10.1038/s41467-022-33863-z (PMC9616875; doi:10.1038/s41467-022-33863-z)
Supplement: Supplementary file 1 — Supplementary information [file 41467_2022_33863_MOESM1_ESM.pdf]

Supplementary Information

A doxycycline- and light-inducible Cre recombinase mouse model for optogenetic genome editing

Miguel Vizoso[1*], Colin E.J. Pritchard[2], Lorenzo Bombardelli[3], Bram van den Broek[4,5], Paul Krimpenfort[2], Roderick L. Beijersbergen[3,6], Kees Jalink[4,7] and Jacco van Rheenen[1**]

[1]Department of Molecular Pathology, Oncode Institute, Netherlands Cancer Institute, Amsterdam, 1066 CX, The Netherlands

[2]Mouse Clinic for Cancer and Aging, The Netherlands Cancer Institute, Amsterdam, 1066 CX, The Netherlands

[3]Division of Molecular Carcinogenesis and Oncode Institute, Netherlands Cancer Institute, Amsterdam, 1066 CX, The Netherlands

[4]Cell Biophysics Group, Department of Cell Biology, The Netherlands Cancer Institute, Amsterdam, The Netherlands

[5]BioImaging Facility, The Netherlands Cancer Institute, Amsterdam, The Netherlands

[6]NKI Robotics and Screening Center and Genomics Core Facility, The Netherlands Cancer Institute, Plesmanlaan 121, Amsterdam, The Netherlands

[7]Swammerdam Institute for Life Sciences, University of Amsterdam, Amsterdam, The Netherlands

*Correspondence: m.vizoso.patino@gmail.com

**Correspondence and lead contact: j.v.rheenen@nki.nl

a

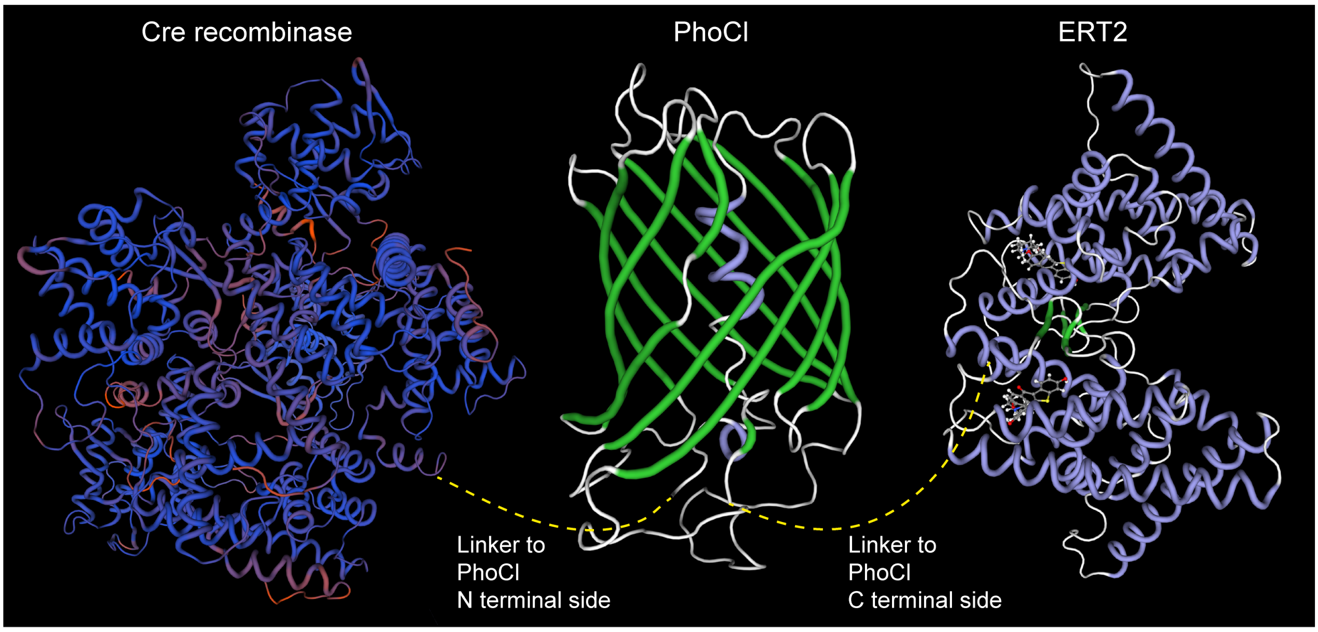
Cre recombinase
PhoCl
ERT2
Linker to PhoCl N terminal side
Linker to PhoCl C terminal side

b

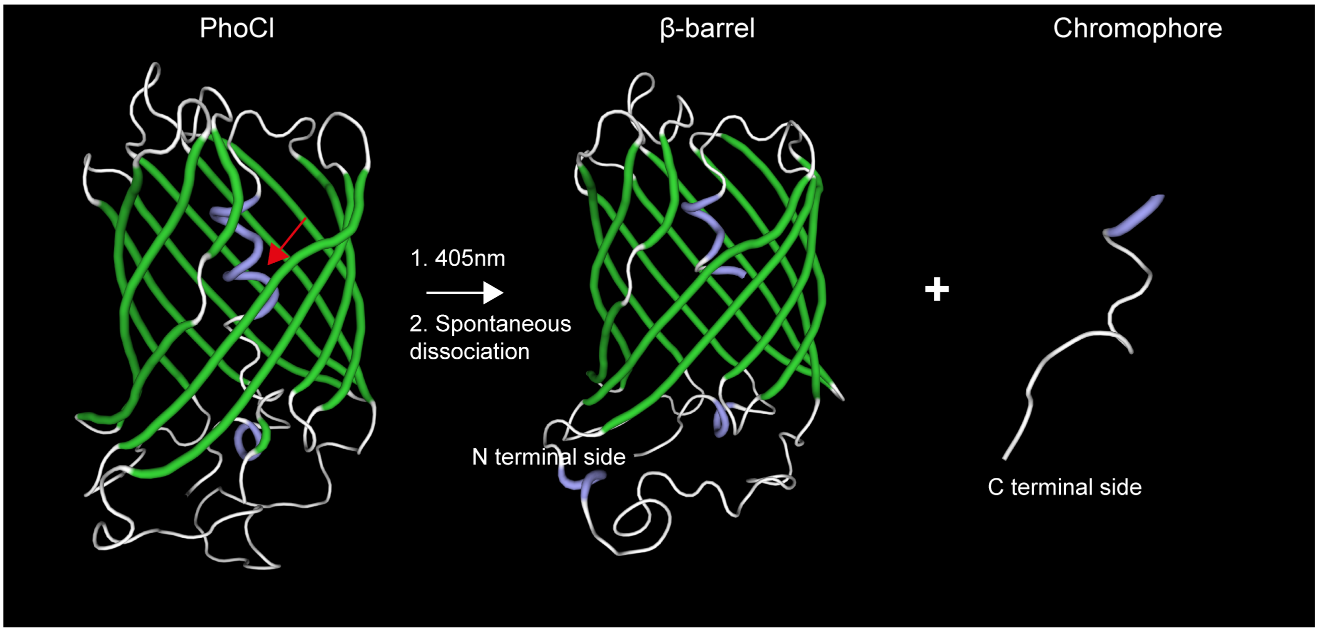
PhoCl
β-barrel
Chromophore
1. 405nm
2. Spontaneous dissociation
+
N terminal side
C terminal side

c

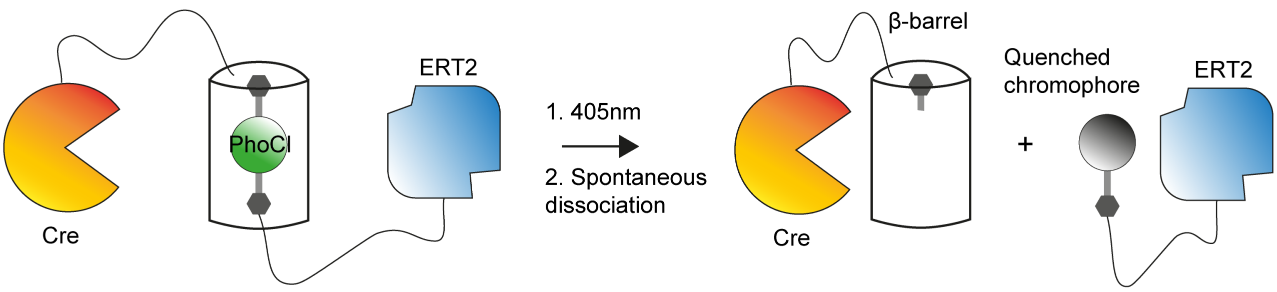
ERT2
PhoCl
Cre
1. 405nm
2. Spontaneous dissociation
β-barrel
Quenched chromophore
ERT2
+
Cre

Supplementary Figure 1: Schematic representation of PhoCl cleavage and Cre recombinase release from the ERT2 domain.

a, DiLiCre1.0 protein depicted in its units which are represented as tertiary protein structures. Tertiary protein structures for Cre recombinase, PhoCl unit, and ERT2 domain were modelled using SWISS-MODEL workspace (Waterhouse et al., 2018).

b, Mechanism of PhoCl cleavage and break-off point (red arrow) by exposure to 405 nm light.

c, Schematic representation of DiLiCre1.0 construct and Cre recombinase release from the ERT2 domain upon 405 nm light illumination. After exposure the Cre recombinase remains attached to the β-barrel of the PhoCl unit but is excised from the ERT2 domain which takes away the quenched chromophore.

Supplementary Figure 2

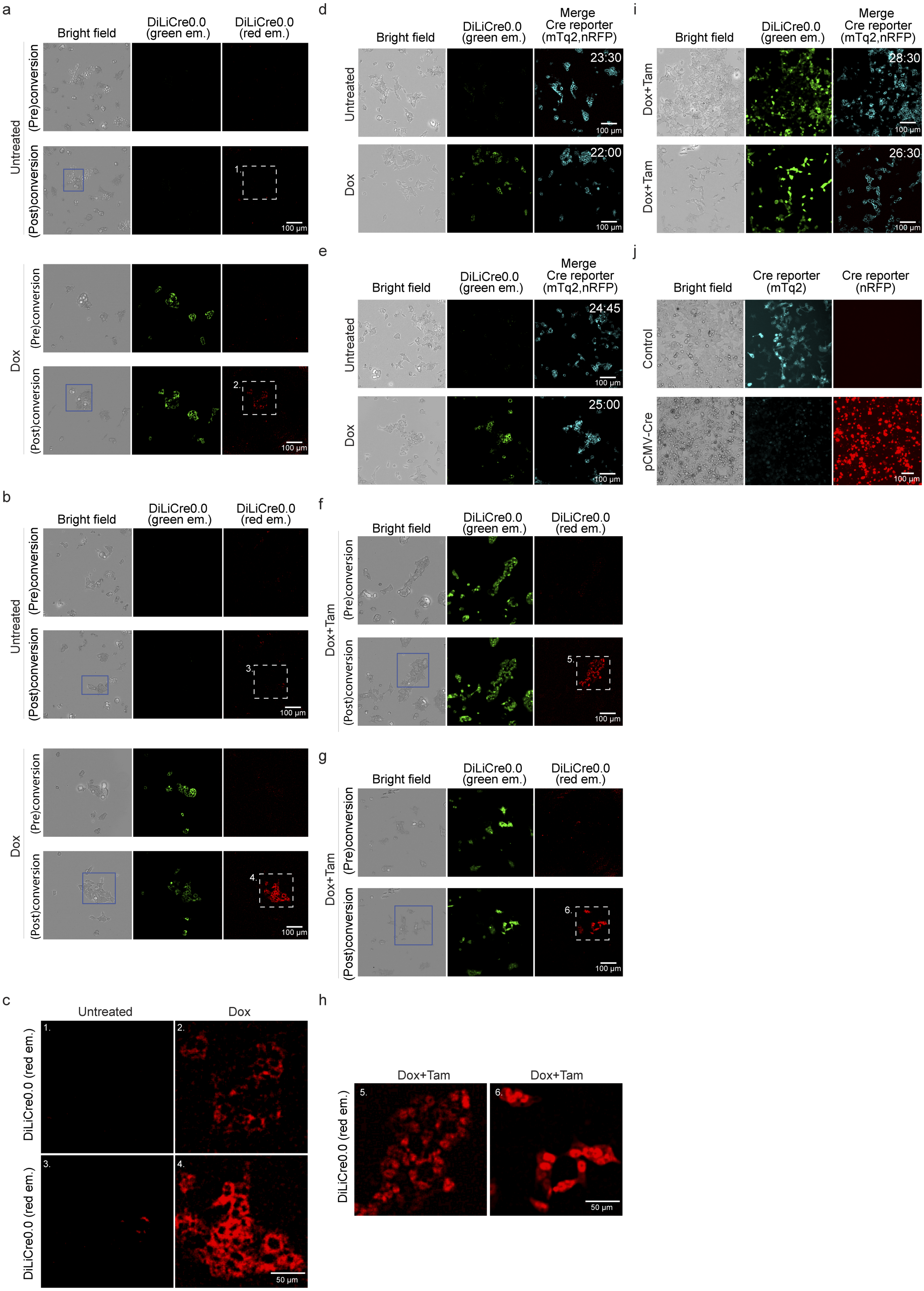

Supplementary Figure 2: Performance of DiLiCre0.0 system following confocal photoactivation and time-lapse imaging

a-b, Representative confocal images of HEK293T cells stably expressing DiLiCre0.0 and memMTQ2-nRFP reporter and exposed to doxycycline. Black boxed areas represent the cell clusters exposed to 405 nm light (1.6 mW/mm^2 on/off pulses of 15 s [x12] in panel **a** and 180 s [x2] in panel **b**). Untreated cells are also shown. White dotted boxed areas represent the photoconverted regions which are magnified in panel **c**. This experiment was independently repeated n=2 times with similar results.

c, Detailed zoom-ins of images shown in **Supplementary Fig. 2a** and **b**.

d-e, Representative endpoint images of time-lapse experiments shown in **Supplementary Fig. 2a** and **b**, respectively. The numbers inside the images represent hours and minutes (hh:mm).

f-g, Representative confocal images of HEK293T cells stably expressing DiLiCre0.0 and memMTQ2-nRFP reporter and exposed to doxycycline and tamoxifen. Black boxed areas represent the cell clusters exposed to 405 nm single photon light (1.6 mW/mm^2 on/off pulses of 15 s [x12] in panel **f** and 180 s [x2] in panel **g**). Untreated cells are also shown. White dotted boxed areas represent the photoconverted regions which are magnified in panel **h**. This experiment was independently repeated n=2 times with similar results

h, Detailed zoom-ins of images shown in **Supplementary Fig. 2f** and **g**.

i, Representative endpoint images of time-lapse experiments shown in **Supplementary Fig. 2g**. The numbers inside the images represent hours and minutes (hh:mm).

j, Representative image of memMTQ2-nRFP Cre reporter recombination upon pCMV-Cre transient transfection in HEK293T cells. This experiment was performed once.

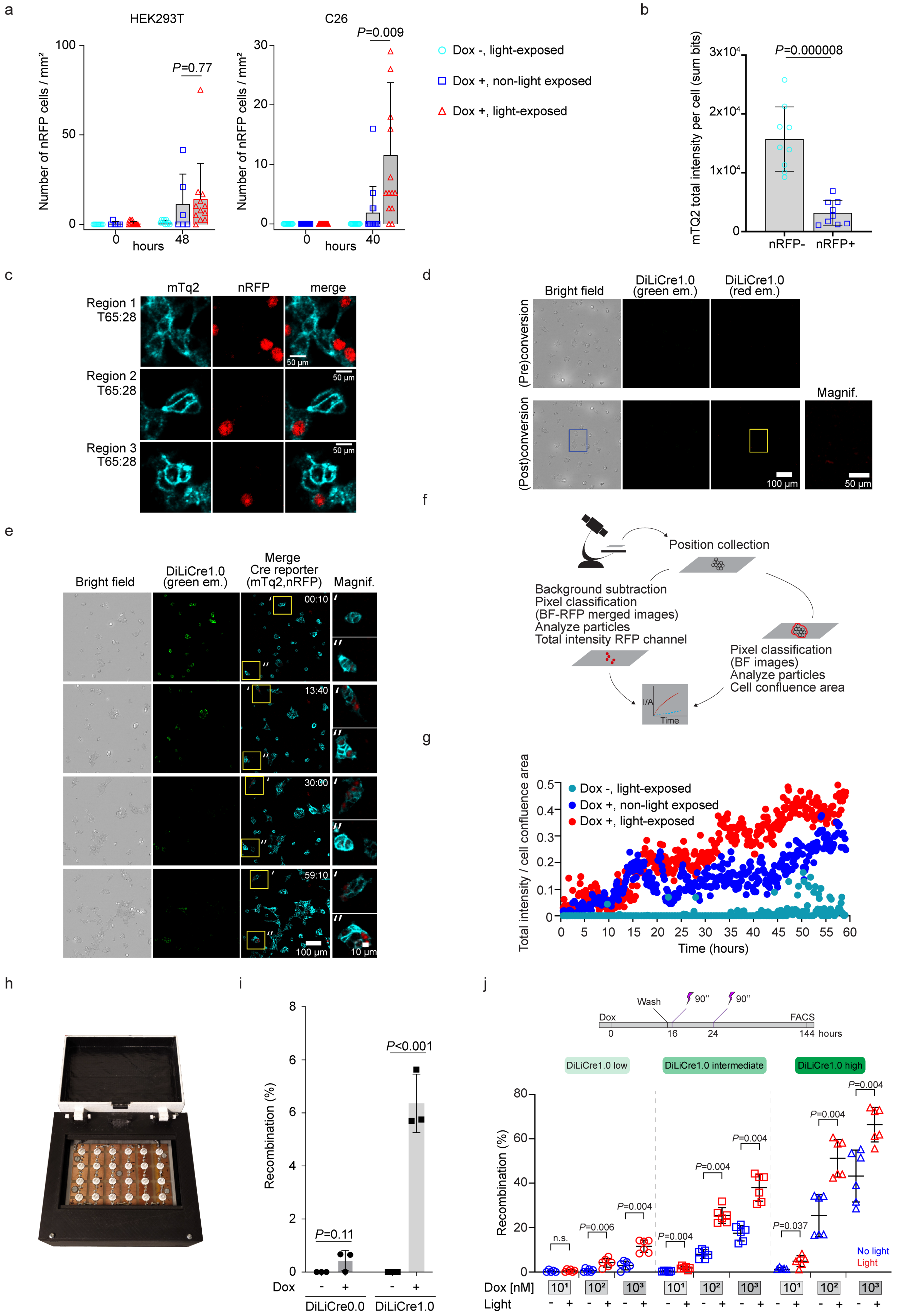
a
HEK293T
C26
Number of nRFP cells / mm²
P=0.77
P=0.009
hours
Dox -, light-exposed
Dox +, non-light exposed
Dox +, light-exposed
b
mTQ2 total intensity per cell (sum bits)
P=0.000008
nRFP-
nRFP+
c
mTq2
nRFP
merge
Region 1 T65:28
Region 2 T65:28
Region 3 T65:28
50 µm
d
Bright field
DiLiCre1.0 (green em.)
DiLiCre1.0 (red em.)
Magnif.
(Pre)conversion
(Post)conversion
100 µm
50 µm
e
Bright field
DiLiCre1.0 (green em.)
Merge Cre reporter (mTq2,nRFP)
Magnif.
00:10
13:40
30:00
59:10
100 µm
10 µm
f
Position collection
Background subtraction
Pixel classification
(BF-RFP merged images)
Analyze particles
Total intensity RFP channel
Pixel classification
(BF images)
Analyze particles
Cell confluence area
I/A
Time
g
Total intensity / cell confluence area
Dox -, light-exposed
Dox +, non-light exposed
Dox +, light-exposed
Time (hours)
h
i
Recombination (%)
P=0.11
P<0.001
Dox
DiLiCre0.0
DiLiCre1.0
j
Wash
90''
Dox
FACS
0
16
24
144 hours
DiLiCre1.0 low
DiLiCre1.0 intermediate
DiLiCre1.0 high
Recombination (%)
n.s.
P=0.006
P=0.004
P=0.037
No light
Light
Dox [nM]
Light
10¹
10²
10³

Supplementary Figure 3: Recombination efficiency of DiLiCre1.0 system in HEK293T cells following time-lapse microscopy and FACS analysis

a, Quantification of the number of nRFP positive cells per mm^2 in HEK293T and C26 cell line models (related to **Figure 1d**). Data points represent the mean±SD obtained from n=6 independent positions examined over n=2 biologically independent experiments. The statistical *P* values were determined by a two-sided t-test.

b, Quantification of memMTQ2 fluorescence intensity per cell comparing nRFP negative and positive cells. Data was obtained from 3 independent imaging positions and measuring three cells per imaging position (related to **Figure 1d**). Data points represent the mean±SD. The statistical *P* values were determined by a two-sided t-test.

c, Representative images of memMTQ2 fluorescence decay in HEK293T cells upon DiLiCre1.0 mediated recombination (related to **Figure 1d**). Data points represent the mean±SD. This experiment was independently repeated n=2 times with similar results.

d, Representative images of HEK293T cells stably expressing DiLiCre1.0 and memMTQ2-nRFP and non-treated with doxycycline. The cell cluster exposed to 405 nm laser light is boxed by a blue square. A detailed magnification of the cells which fail to photoconverted is shown (yellow square).

e, Representative time-lapse confocal images of HEK293T cells stably expressing DiLiCre1.0 and memMTQ2-nRFP and treated with doxycycline. Detailed magnifications of random recombinant clusters are shown (yellow squares). The numbers inside the images represent hours and minutes (hh:mm). This experiment was independently repeated n=2 times with similar results.

f, Schematic drawing of the workflow analysis followed to quantify the recombination rate during confocal time-lapse experiments.

g, Recombination levels related to panel **f** and representing the total RFP intensity divided by the area of cell confluence. Data points represent the mean±SEM. n=2 biologically independent experiments were performed with similar results.

h, 3D printed 24-array (adjustable) LED box used for whole well photoactivation.

i, FACS data quantification of light-mediated recombination in HEK293T cells stably expressing DiLiCre0.0 or DiLiCre1.0 and treated with 100 nM for 24 hours. Data points represent the mean±SD obtained from n=3 biologically independent experiments. The statistical *P* values were determined by a two-sided t-test.

j, FACS quantification of DiLiCre1.0-mediated recombination in HEK293T cells stably expressing the memMTQ2-nRFP reporter and sorted for low, intermediate and high DiLiCre1.0 expression. The three sorted populations were tested for three different concentrations of doxycycline (10, 100, or 1000 nM). Control values (untreated cells) were subtracted. Within each sorted population, doxycycline treated cells were both exposed (0.5 mW/mm^2 90 s-pulses every 4 hours [x2], red data points) and non-exposed to light (blue data points). Data represents the mean±SD of 6 biologically independent experiments. The statistical *P* values were determined by Mann-Whitney test.

Source data are provided as a Source Data file.

Supplementary Figure 4

a

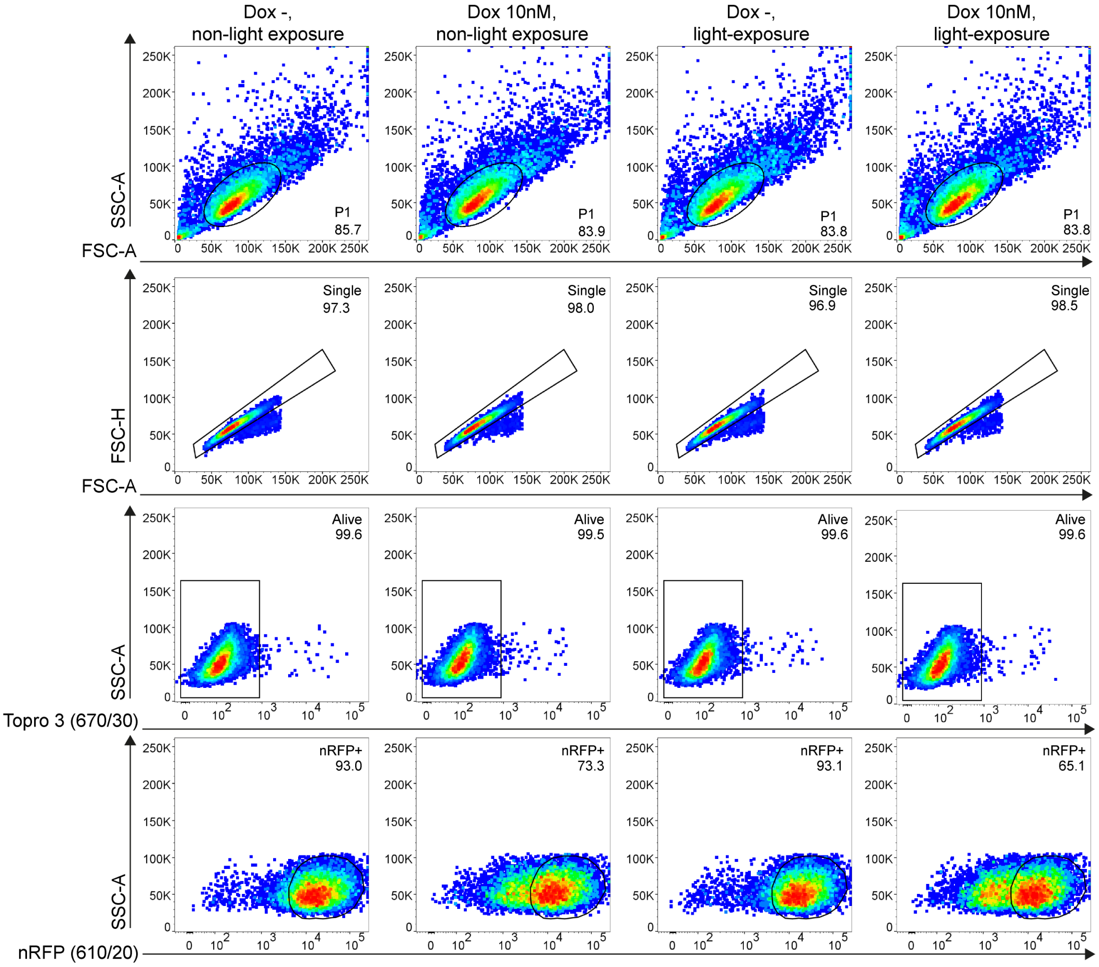

b

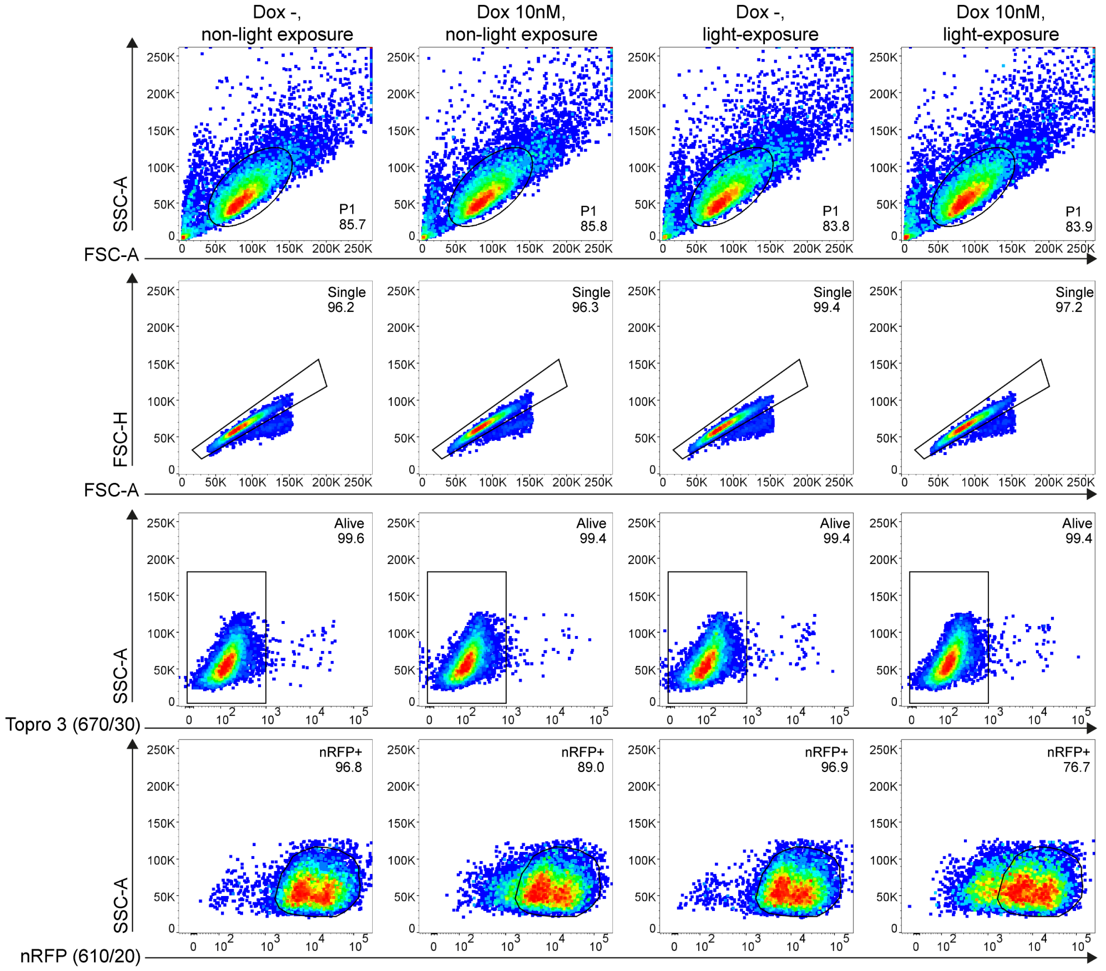

Supplementary Figure 4: Raw FACS data corresponding to Figure 2d.

a, Representative FACS plots for the evaluation of light-mediated recombination in HEK293T cells transduced with DiLiCre1.0 and a red nuclear Cre reporter. Four different conditions were tested: untreated cells, cells exposed to doxycycline or light, and combined treatment (10 nM dox and 0.5 mW/mm^2 30 s-pulses every 4 hours [x3]).

b, Representative FACS plots for the evaluation or light-mediated recombination in HEK293T cells transduced with DiLiCre2.0 and a red nuclear Cre reporter. Four different conditions were tested: untreated cells, cells exposed to doxycycline or light, and combined treatment (10 nM dox and 0.5 mW/mm^2 30 s-pulses every 4 hours [x3]).

Supplementary Figure 5

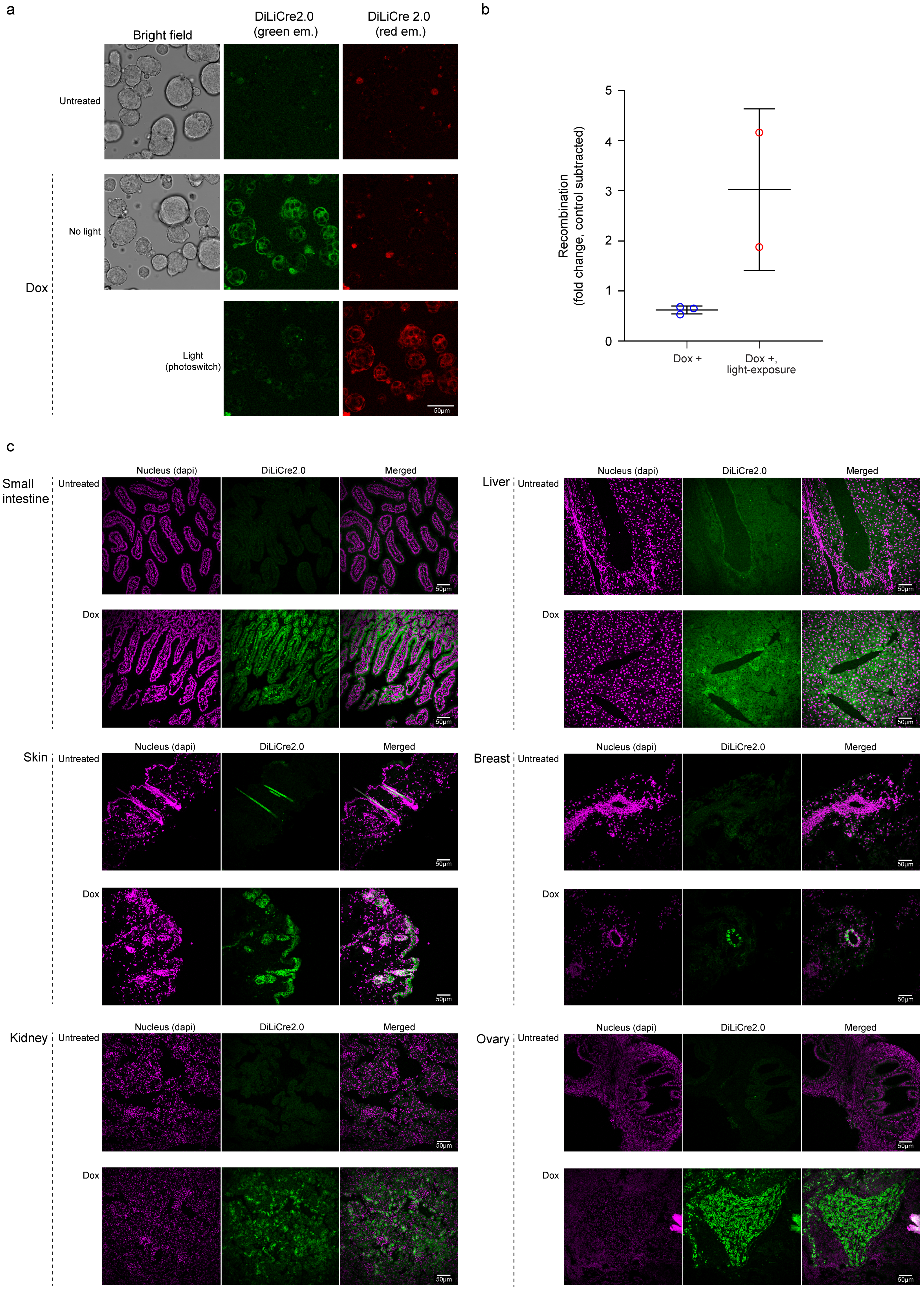

Supplementary Figure 5: DiLiCre2.0 system characterization in mESCs and adult tissues

a, Representative images of DiLiCre2.0 expression and photoconversion in B6 (C57BL/6J) mESCs cells revealed by confocal microscopy. This experiment was independently repeated n=2 times with similar results.

b, FACS data quantification of DiLiCre2.0 mESCs transduced with a floxable nuclear RFP cassette. Data represents mean±SD from n=3 biologically independent experiments.

c, Representative images of tissue sections from different organs collected from 8-week old DiLiCre2.0 transgenic mice treated with doxycycline (1mg per 25g of body weight) for 24hours. Images shown the endogenous cytoplasmic fluorescence of DiLiCre2.0 (green) and nuclei staining (magenta) as separated and merged channels. This experiment was performed once.

Source data are provided as a Source Data file.

a

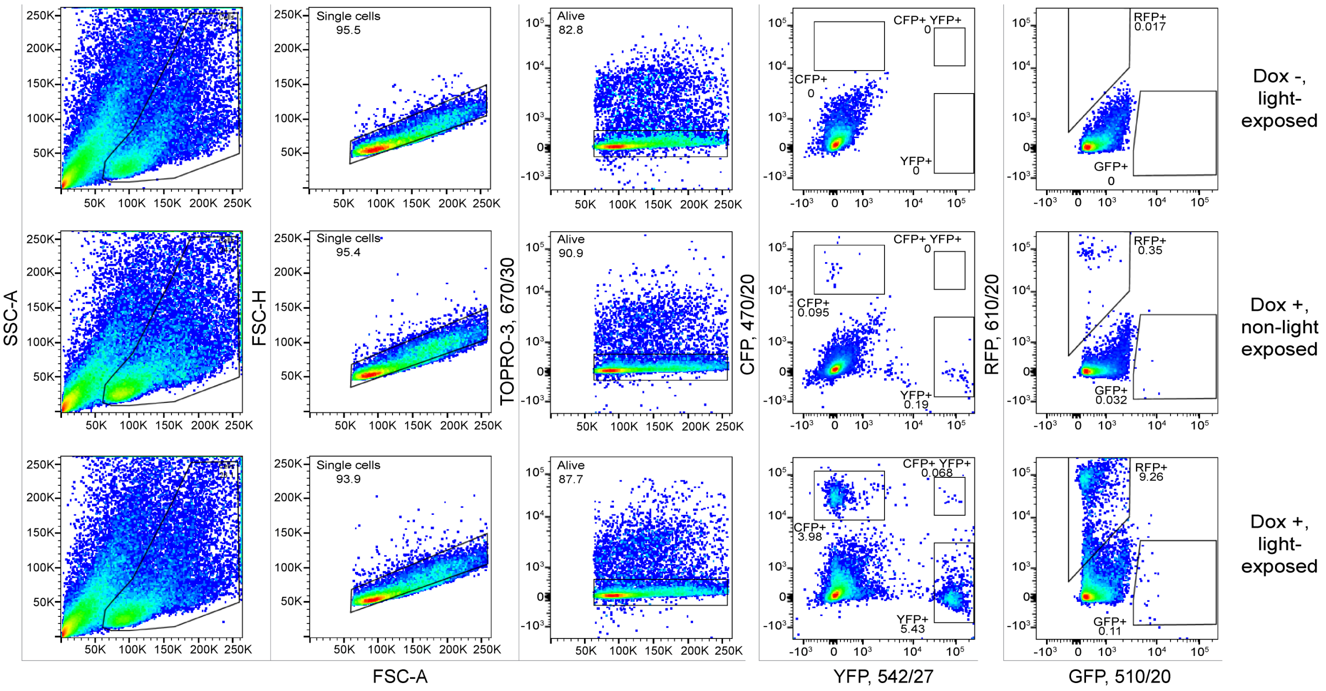

b

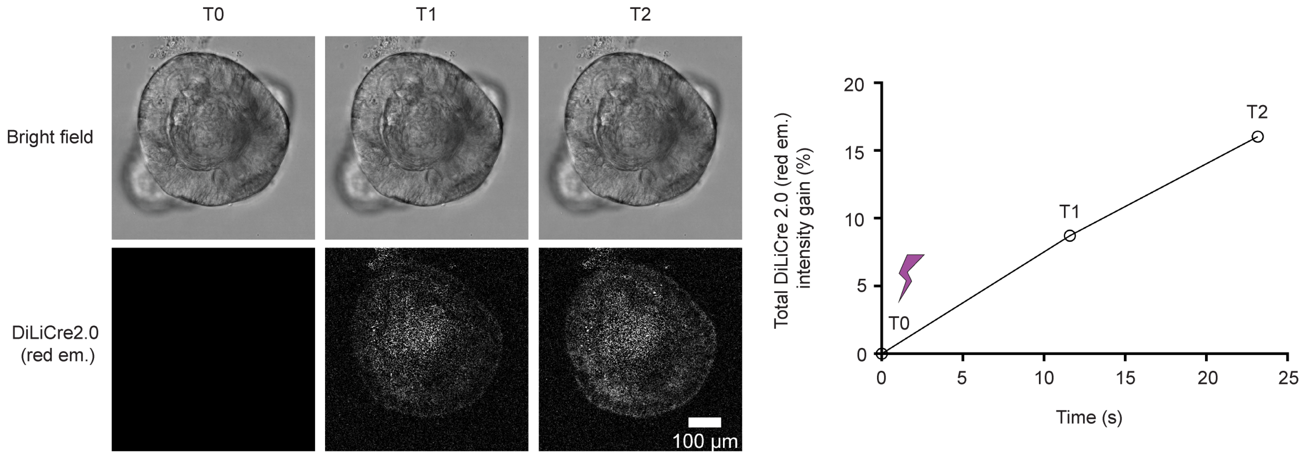

c

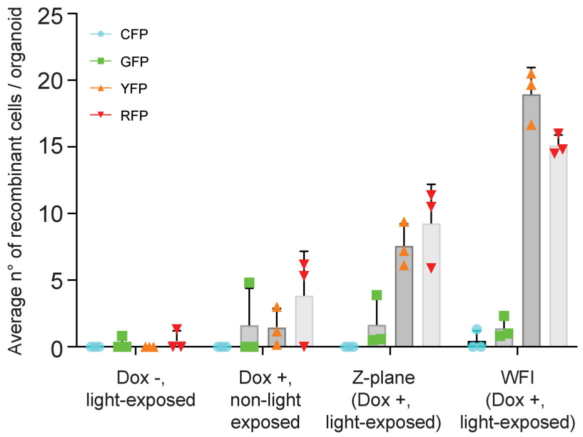

	P values	Dox -, light-exposed	Dox +, non-light exposed	Z-plane (Dox +, light-exposed)	WFI (Dox +, light-exposed)
YFP	Light-exposed		n.s.	0.002	<0.001
	Dox+	n.s.		0.006	<0.001
	Z-plane	0.002	0.006		<0.001
	WFI	<0.001	<0.001	<0.001	
RFP	Light-exposed		n.s.	0.009	<0.001
	Dox+	n.s.		n.s.	0.002
	Z-plane	0.009	n.s.		n.s.
	WFI	<0.001	<0.002	n.s.	

Supplementary Figure 6: ***Ex vivo*** **photoconversion of small intestine organoids derived from DiLiCre2.0;R26-Confetti mice**

a, Representative FACS plots for the evaluation of light-mediated recombination in intestinal organoids *ex vivo*. Organoids were treated with doxycycline (10, 50 or 100 nM) and/or exposed to 405 nm LED light (0.5 mW/mm^2 30 s-pulses every 4 hours [x3]). n=3 biologically independent replicates were performed with similar results.

b, Confocal representative images and quantification of the DiLiCre2.0 transient photoconversion (green-to-red) and response to 405 nm laser light. n=3 biologically independent replicates were performed with similar results.

c, Plot summarizing the average number of (C/G/Y/R)FP recombinant cells per small intestinal organoid upon photoactivation (related to **Fig. 4c** and **d**). Organoids were imaged for approximately 40 hours and quantification was performed at the last taken frame. Data represents the mean±SD of n=3 biologically independent experiments. To determine the *P* values, Bonferroni method was applied to post hoc multiple comparisons after one-way anova.

Source data are provided as a Source Data file.

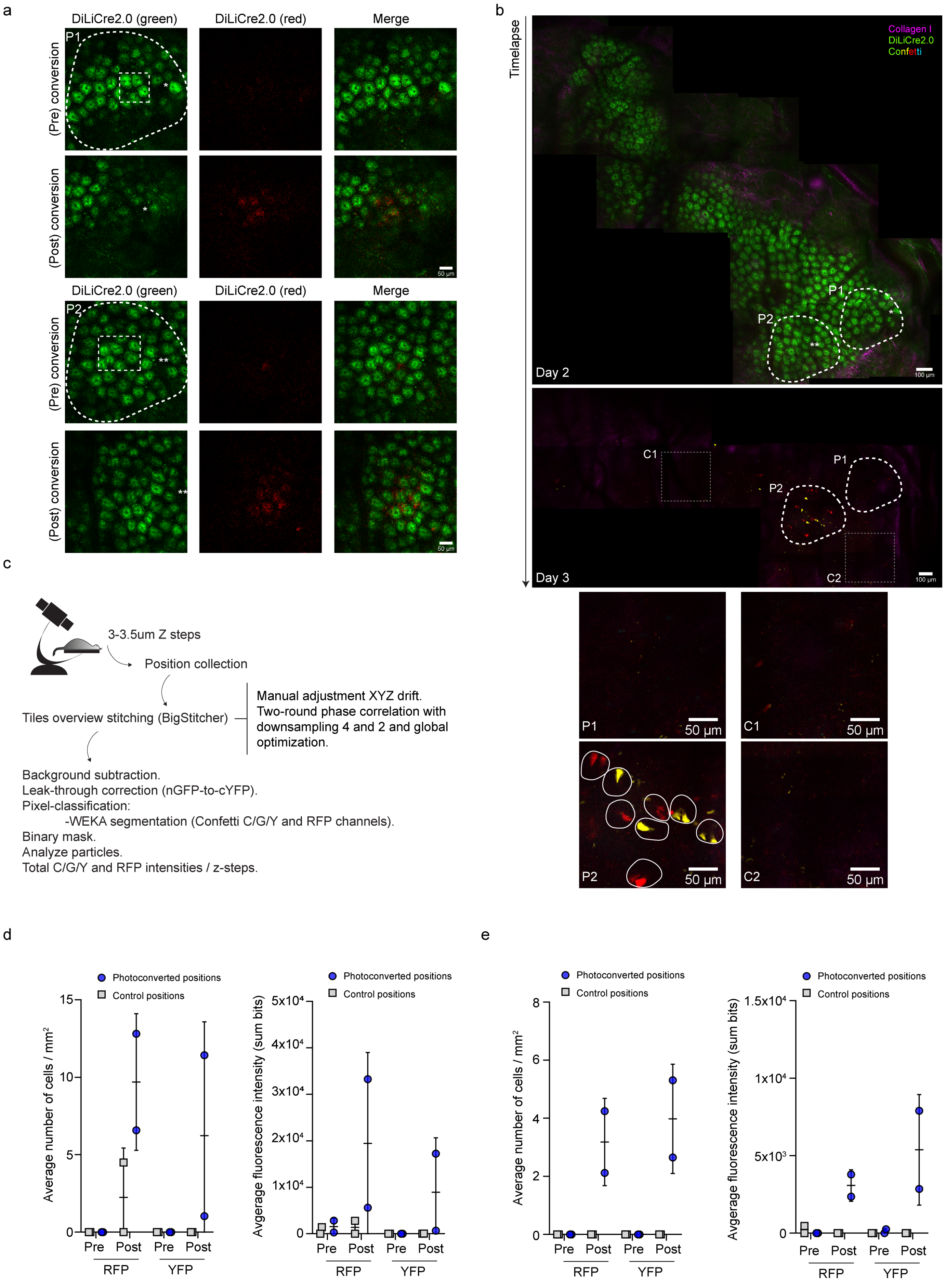
a
DiLiCre2.0 (green)
DiLiCre2.0 (red)
Merge
(Pre) conversion
(Post) conversion
P1
P2
50 µm
b
Timelapse
Collagen I
DiLiCre2.0
Confetti
Day 2
Day 3
P1
P2
C1
C2
100 µm
50 µm
c
3-3.5um Z steps
Position collection
Tiles overview stitching (BigStitcher)
Manual adjustment XYZ drift.
Two-round phase correlation with downsampling 4 and 2 and global optimization.
Background subtraction.
Leak-through correction (nGFP-to-cYFP).
Pixel-classification:
-WEKA segmentation (Confetti C/G/Y and RFP channels).
Binary mask.
Analyze particles.
Total C/G/Y and RFP intensities / z-steps.
d
e
Photoconverted positions
Control positions
Average number of cells / mm²
Avgerage fluorescence intensity (sum bits)
Pre
Post
RFP
YFP

Supplementary Figure 7: Two-multiphoton longitudinal assay in the DiLiCre2.0 mice revealed photoswitching of cells in intestinal crypt areas with low background recombination

a, Two intestinal ROIs are represented (P1 and P2) where zoom-in areas (white boxed regions) were exposed to 405 nm laser and photoconversion. White asterisks on the picture were placed in order to help the reader to recognize the same structures in the longitudinal sequential images shown in the following panels. Gaussian filter 1.5 applied for visualization. This experiment was independently repeated n=3 times with similar results.

b, Longitudinal two-photon overviews of the intestine containing the photoconverted areas annotated in panel (**a**). Detailed magnifications from both ROIs and control regions are also shown. Gaussian filter 1 applied for visualization.

c, Quantification strategy followed to measure Confetti fluorescence intensities.

d-e, Quantifications of RFP and YFP cell numbers and fluorescence intensities pre- and 24 hours post-photoconversion (~24 hours post-conversion). Measurements were performed in the photoconverted and non-photoconverted areas. Same square dimensions were used for all measured positions (225x225 μm). Control positions were selected randomly at least 150 μm away from the photoconverted areas. Data represents mean ±SD. n=3 biologically independent experiments/mice were performed with similar results.

Source data are provided as a Source Data file.

Supplementary Figure 8

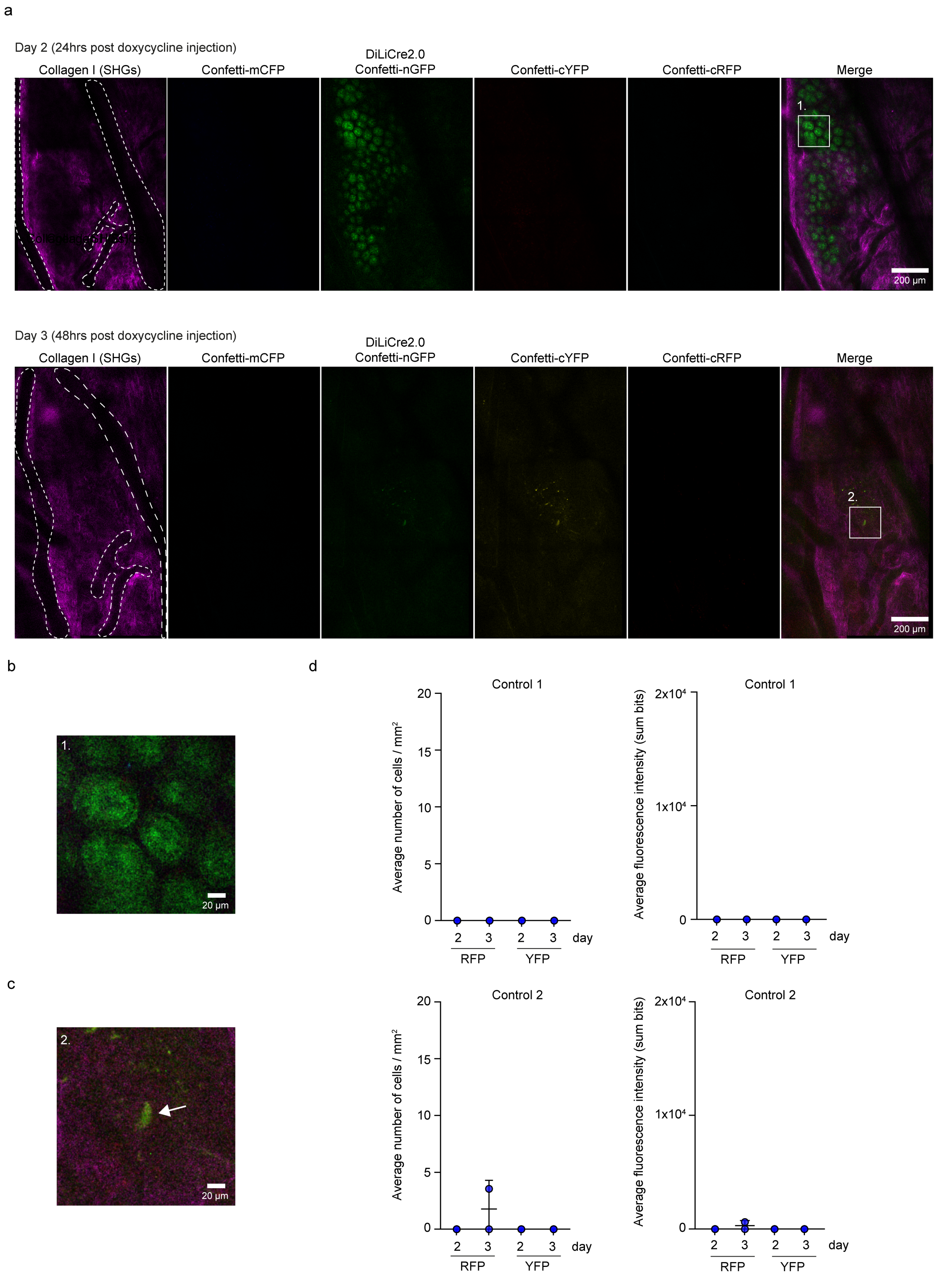

Supplementary Figure 8: Levels of background recombination in the intestine in the *DiLiCre2.0;R26-Confetti* mouse model.

a, Tissue overviews representing the small intestinal area scanned by two-photon microscopy 24 hours and 48 hours post-doxycycline treatment, top and bottom sequences, respectively. White dotted outlines are shown in the Collagen I (SHGs) overviews to help the reader to recognize the same structures at different days. DiLiCre2.0 positive crypts (box 1) and one Confetti recombinant cell (box 2) are highlighted using white boxes.

b, Zoom-in of the region annotated in panel **a** (upper row).

c, Zoom-in of the region annotated in panel **a** (bottom row). An arrow is used to highlight one Confetti recombinant cell.

d, Quantification of cYFP and cRFP Confetti fluorescence intensities and cell numbers 24 and 48 hours post-doxycycline treatment (days 2 and 3, respectively). All biological replicates shown similar results. Data represents the mean±SD from n=2 independent positions examined over n=2 biologically independent experiments.

Source data are provided as a Source Data file.

a

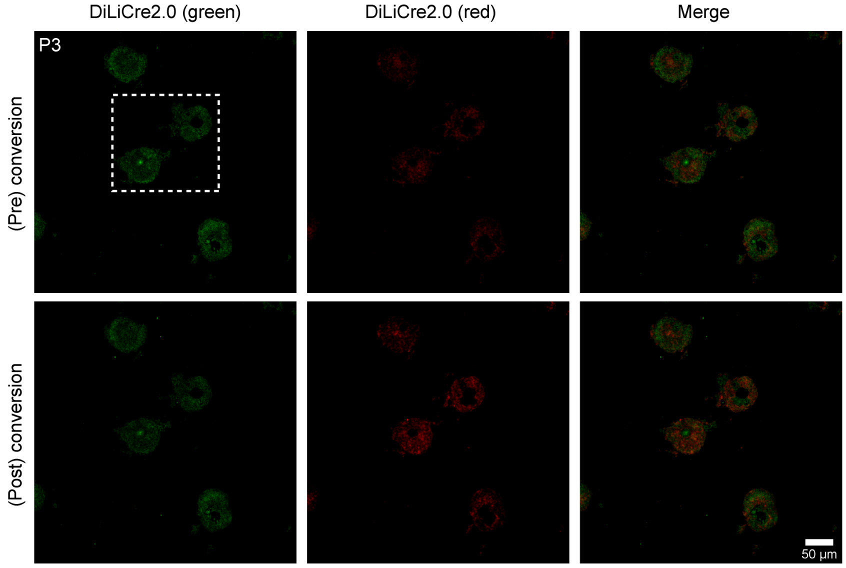

b

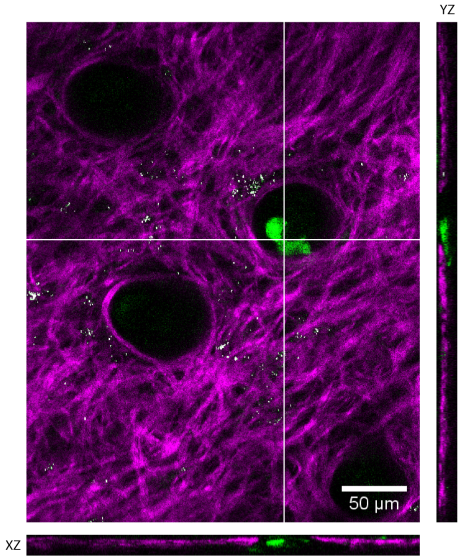

c

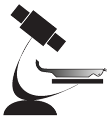

Skin imaging (3.5-4um Z steps)

Position collection

Tile stiching (Leica LAS X stitching 3D module) and XY drift correction (Big-warp and manual landmark annotation).
Channel leak-through correction (DiLiCre2.0-HrasV12 into red (”empty channel”)) and image subtraction (red to DiLiCre2.0-HrasV12 channel).
Fig. 5d: Measuring green total intensity.
Total intensity / z-steps.
Fig. 6c: Measuring green total intensity (HrasV12) and SHGs (collagen I) total intensities per z-plane.

d

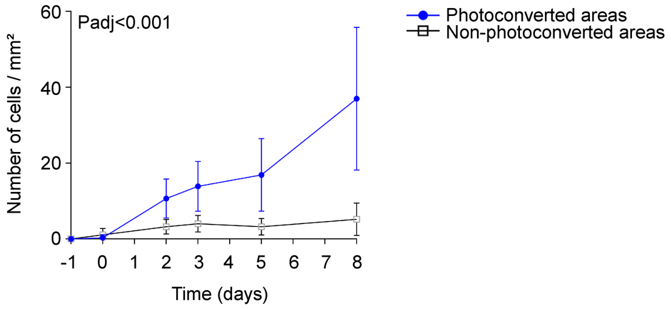

Supplementary Figure 9: Photoconversion of the DiLiCre2.0 system in the mouse skin

a, Photoconversion of DiLiCre2.0 in HFs of *DiLiCre2.0;HrasV12-eGFP* animals (position 3). Gaussian filter 1.5 applied for visualization.

b, Two multiphoton representative image 48 hours post-activation showing a small cluster of HrasV12 recombinant cells. The image correspond to the same position shown in panel **a**. Orthogonal views (XZ and YZ) from same Z stack are shown evidencing the presence of HrasV12 recombinant cells within the HF.

c, Quantification strategy followed to measure the HrasV12 recombination in longitudinal experiments of the skin.

d, Quantification of HrasV12-eGFP cell numbers per mm2 comparing 405 nm photoconverted and non-photoconverted areas in the skin (related to **Figure 5c** and **d**). Data summarizes the results obtained from n=3 independent biological replicates. Data represents the mean±SD. Entire curves were compared statistically and the *P* values were determined by permutation test adapted from Elso *et al*., (2004).

Source data are provided as a Source Data file.

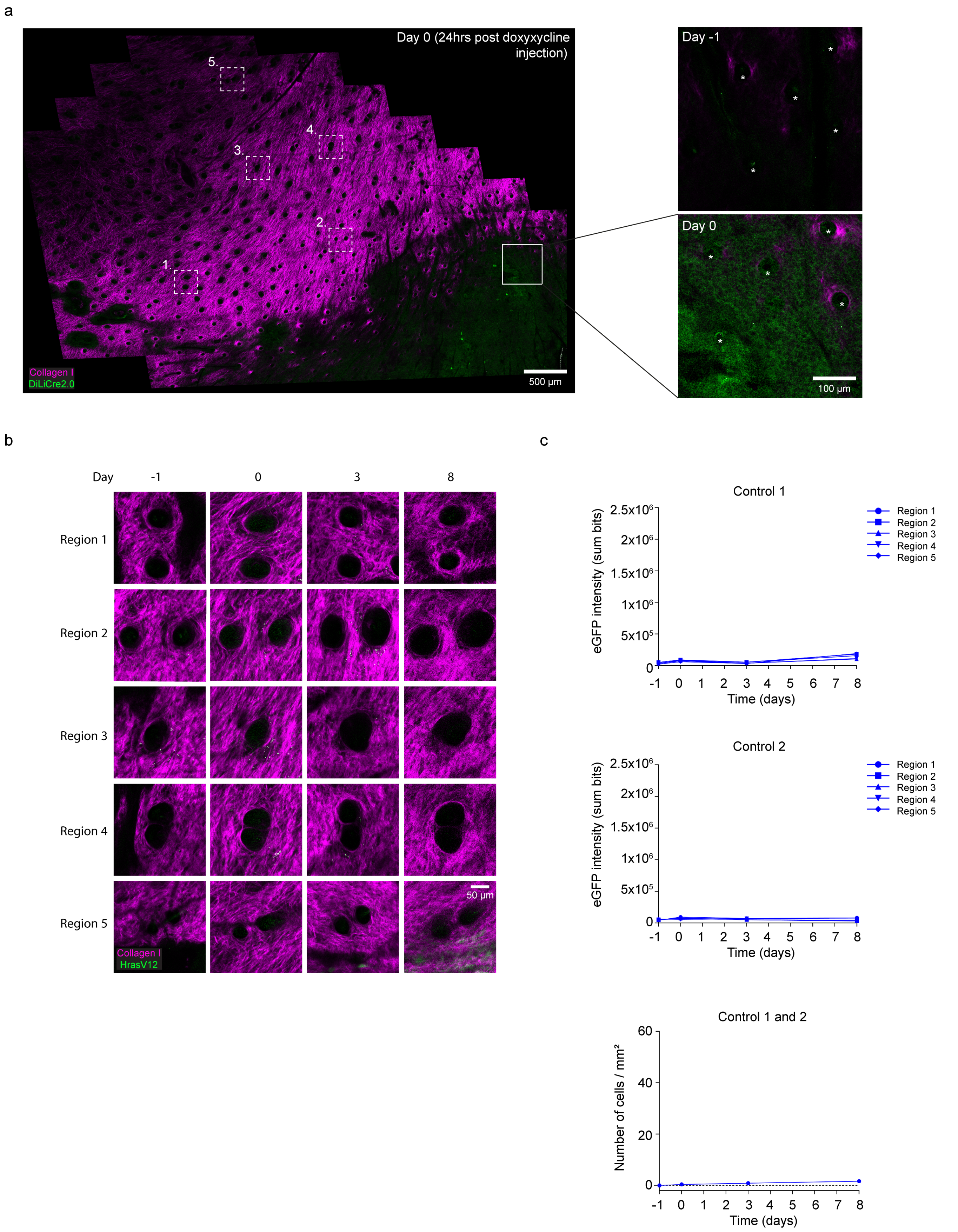
a
Day 0 (24hrs post doxyxycline injection)
5.
4.
3.
2.
1.
Collagen I
DiLiCre2.0
500 µm
Day -1
Day 0
100 µm
b
Day
-1
0
3
8
Region 1
Region 2
Region 3
Region 4
Region 5
50 µm
Collagen I
HrasV12
c
Control 1
eGFP intensity (sum bits)
2.5x10^6
2x10^6
1.5x10^6
1x10^6
5x10^5
0
Time (days)
Region 1
Region 2
Region 3
Region 4
Region 5
Control 2
Control 1 and 2
Number of cells / mm²
60
40
20
0
-1 0 1 2 3 4 5 6 7 8

Supplementary Figure 10: Levels of background recombination in the skin in the *DiLiCre2.0;HrasV12-eGFP* transgenic mouse model.

a, Two-photon overview of the ear skin 24 hours post-doxycycline treatment. Magnification images of the same region (white outlined square) are shown at day of doxycycline treatment (day -1) and 24 hours post-doxycycline treatment (day 0). Five random regions, boxed using white dotted squares, were selected for the longitudinal characterization of the recombinant cells and fluorescence intensity (related to **Supplementary Fig. 10b** and **c** and **Video 5**). This experiment was independently repeated n=2 times with similar results.

b, Representative longitudinal images of the five white dotted squares represented in panel **a**. Two-photon images corresponding to those regions are shown. n=2 biologically independent experiments/mice were performed with similar results.

c, Quantification of HrasV12-eGFP fluorescence intensities and cell numbers within the five white dotted square regions annotated in panel **a** and **b**. All biological replicates shown similar results. Source data are provided as a Source Data file.

Supplementary Figure 11

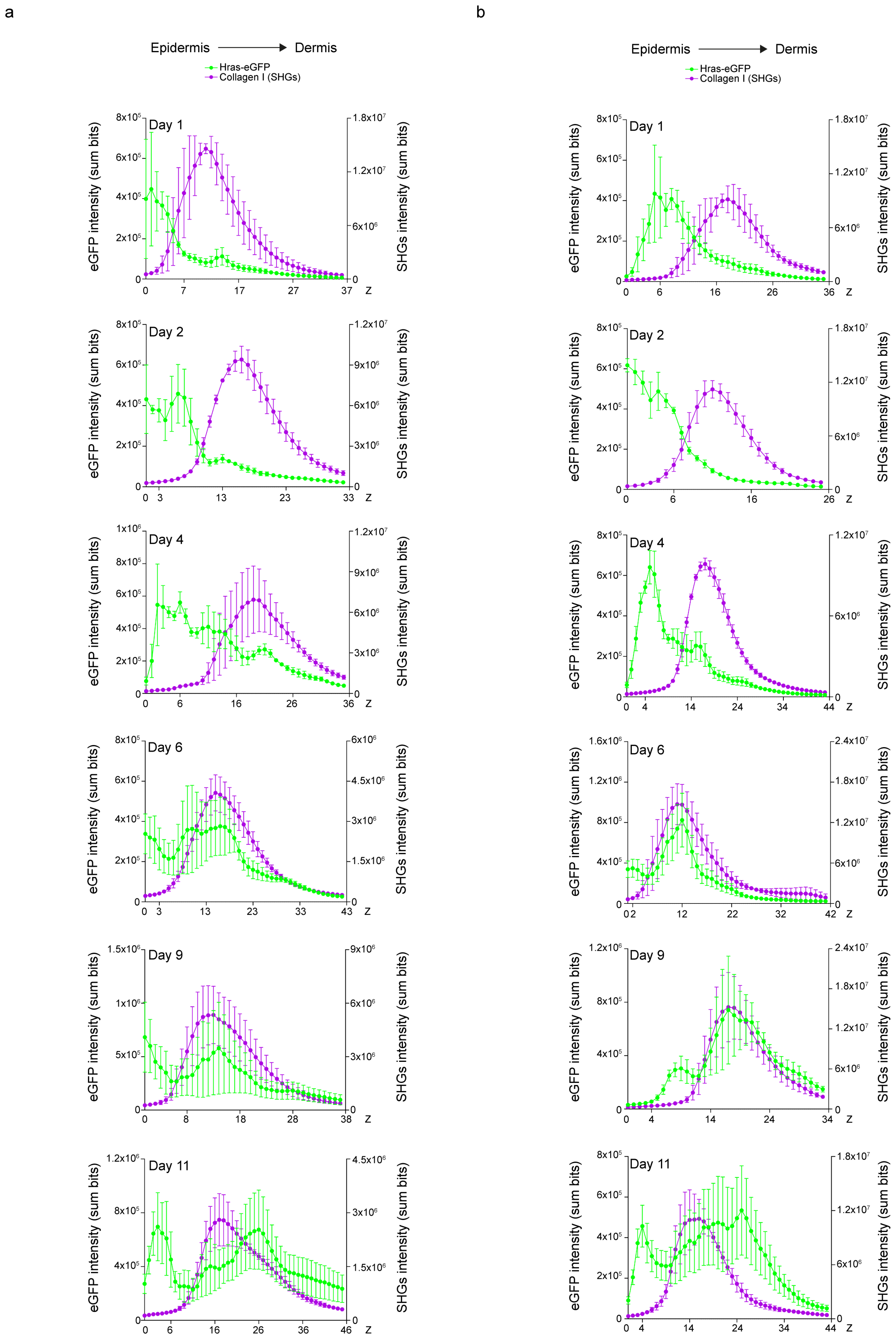

Supplementary Figure 11: DiLiCre2.0-mediated HrasV12 activation in the basal layer of mice skin leads to retrograde movement of cells towards the dermis (replicates 2 and 3)

a, Quantification of HrasV12-eGFP and SHGs (days 1, 2, 4, 6, 9, 11) intensities along the z-axis to identify the position of HrasV12 cells over multiple imaging days (second replicate). Each line represents the average intensities of n=3 photoconverted regions. Data represents mean±SEM.

b, Quantification of HrasV12-eGFP and SHGs (days 1, 2, 4, 6, 9, 11) intensities along the z-axis to identify the position of HrasV12 cells over multiple imaging days (third replicate). Each line represents the average intensities of n=4 photoconverted regions. Data represents mean±SEM.

Source data are provided as a Source Data file.

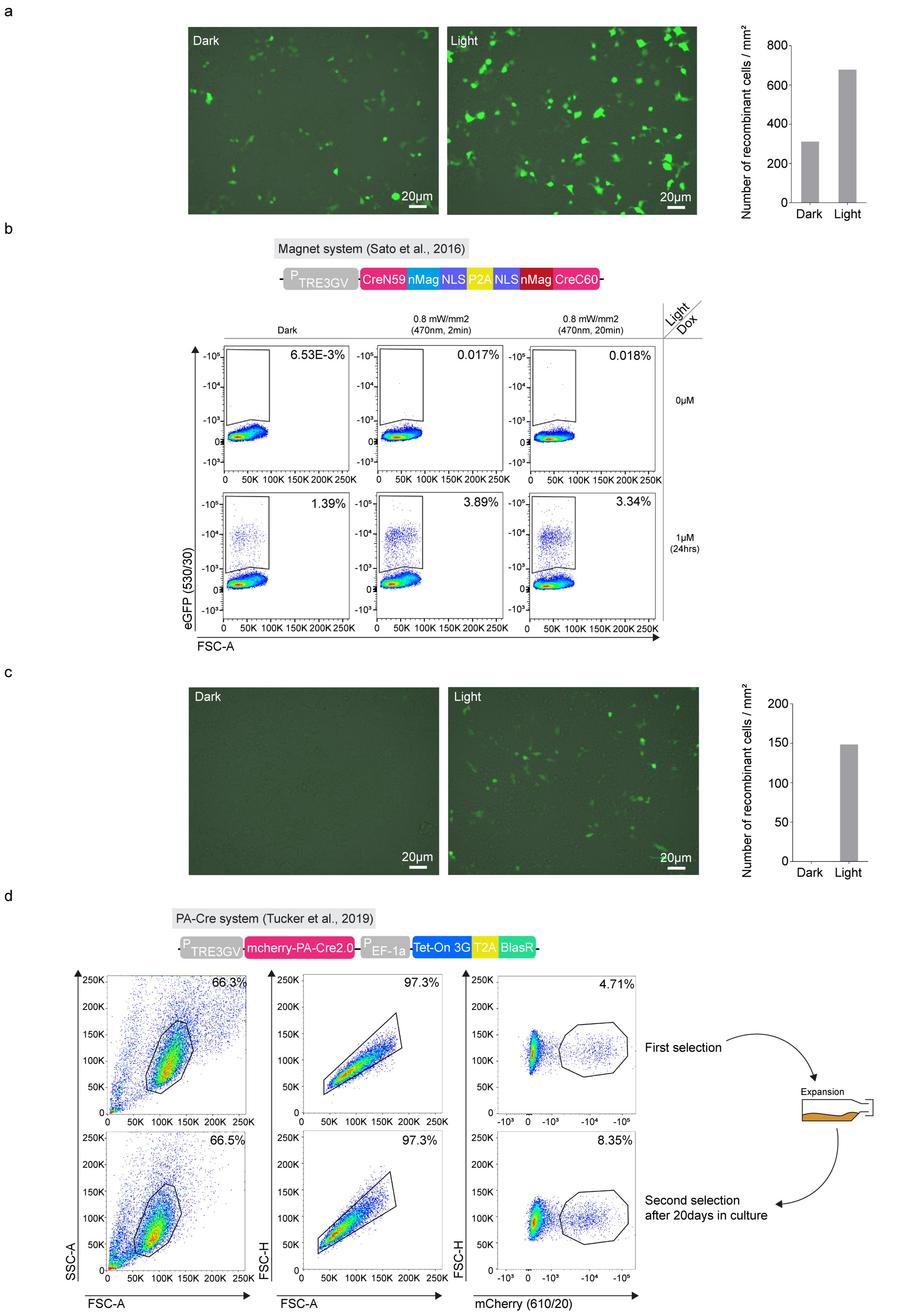
a
Dark
Light
20μm
Number of recombinant cells / mm²
800
600
400
200
0
Dark
Light
b
Magnet system (Sato et al., 2016)
PTRE3GV
CreN59
nMag
NLS
P2A
NLS
nMag
CreC60
Dark
0.8 mW/mm2 (470nm, 2min)
0.8 mW/mm2 (470nm, 20min)
Light
Dox
6.53E-3%
0.017%
0.018%
0μM
1.39%
3.89%
3.34%
1μM (24hrs)
eGFP (530/30)
FSC-A
0
50K
100K
150K
200K
250K
c
Dark
Light
20μm
Number of recombinant cells / mm²
200
150
100
50
0
Dark
Light
d
PA-Cre system (Tucker et al., 2019)
PTRE3GV
mcherry-PA-Cre2.0
PEF-1a
Tet-On 3G
T2A
BlasR
66.3%
97.3%
4.71%
66.5%
97.3%
8.35%
SSC-A
FSC-H
FSC-H
FSC-A
FSC-A
mCherry (610/20)
First selection
Expansion
Second selection
after 20days in culture

Supplementary Figure 12: Performance characterization of the Magnet and PA-Cre2.0 dimerizer systems for the photoactivation of the Cre recombinase.

a, Epifluorescence images of HEK293T cells transiently co-transfected with 0.5 µg of Magnet system (Kawano et al., 2016) and 0.5 µg of Cre reporter. Image on the left represents cells that were kept in the dark and image on the right represents cells exposed to 470 nm light (0.8 mW/mm^2 for 60 s pulse). Epifluorescence images were taken 24 hours post-illumination. Plot on the right represents the images quantification of eGFP cell numbers per square millimeter.

b, Schematic representation of the Magnet photoactivatable Cre recombinase system (adapted from Kawano et al., 2016) containing the tetracycline response element promoter (TRE3GV) and the two catalytically inactive Cre subunits (CreN59 and CreC60). Below, FACS plots for the evaluation of the recombination levels in HEK293T cells stably expressing the Magnet system and the Cre reporter. Cells were keep in the dark or exposed to 470 nm LED light (0.8 mW/mm^2 for 120 s or 20 min pulse) both in the absence or presence of doxycycline (100 nM).

c, Epifluorescence images of HEK293T cells transiently co-transfected with 0.5 µg of PA-Cre2.0 [Meador et al., 2019, CRY2(mutant L348F)-CreN/CIB1(full length)-CreC] and 0.5 µg of Cre reporter. Image on the left represents cells that were kept in the dark and image on the right represents cells exposed to 470 nm light (0.8 mW/mm^2 for 60 s pulse). Epifluorescence images were taken 24 hours post-illumination. Plot on the right represents the images quantification of eGFP cell numbers per square millimeter.

d, Schematic representation of the PA-Cre2.0 photoactivatable Cre recombinase system (adapted from Meador et al., 2019) containing the tetracycline response element promoter (TRE3GV) and the two catalytically inactive Cre subunits (CreN and CreC) in frame with mCherry. Below, FACS strategy for the selection and enrichment of HEK293T cells carrying mCherry upon lentiviral infection with PA-Cre2.0.

Source data are provided as a Source Data file.

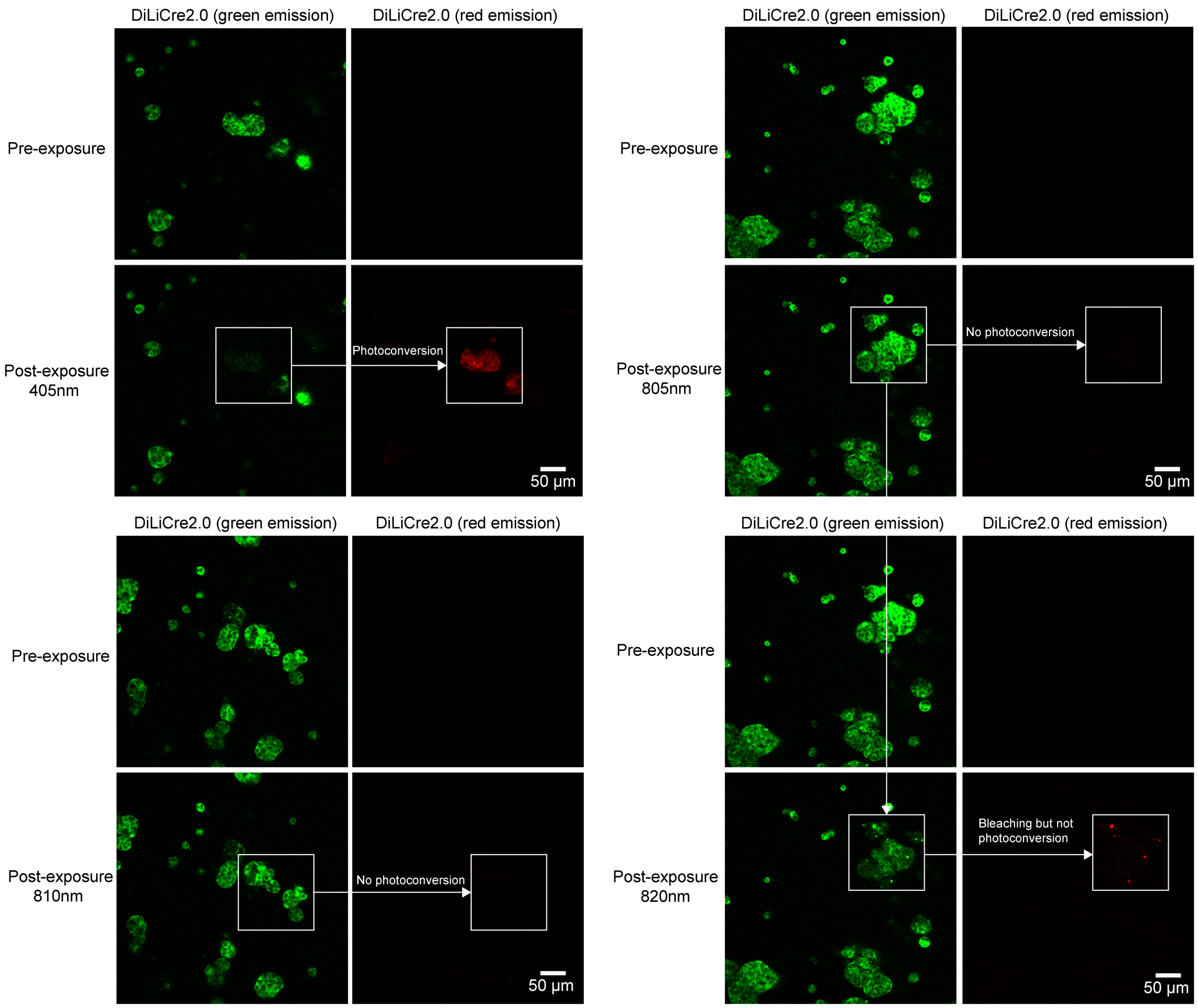
DiLiCre2.0 (green emission)
DiLiCre2.0 (red emission)
Pre-exposure
Post-exposure 405nm
Photoconversion
50 µm
Pre-exposure
Post-exposure 805nm
No photoconversion
50 µm
Pre-exposure
Post-exposure 810nm
No photoconversion
50 µm
Pre-exposure
Post-exposure 820nm
Bleaching but not photoconversion
50 µm

Supplementary Figure 13: Efficiency of two-photon light to photoconvert DiLiCre2.0. Representative confocal images of organoids cultures stably expressing DiLiCre2.0 before and after photoactivation with 405 nm (top left), 805 nm (top right), 810 nm (bottom left), and 820 nm (bottom right). The photoactivated region is boxed by white lines and is mediated by single pulse of laser light.
