# Supplementary material for: A doxycycline- and light-inducible Cre recombinase mouse model for optogenetic genome editing
Source: Nat Commun. 2022 Oct 28;13:6442. doi: 10.1038/s41467-022-33863-z (PMC9616875; doi:10.1038/s41467-022-33863-z)
Supplement: Supplementary file 2 — Reporting Summary [file 41467_2022_33863_MOESM2_ESM.pdf]

nature portfolio

Corresponding author(s): Miguel Vizoso Patiño and Jacco van Rheenen

Last updated by author(s): Sep 30, 2022

Reporting Summary

Nature Portfolio wishes to improve the reproducibility of the work that we publish. This form provides structure for consistency and transparency in reporting. For further information on Nature Portfolio policies, see our Editorial Policies and the Editorial Policy Checklist.

Statistics

For all statistical analyses, confirm that the following items are present in the figure legend, table legend, main text, or Methods section.

n/a	Confirmed	
☐	☒	The exact sample size (n) for each experimental group/condition, given as a discrete number and unit of measurement
☐	☒	A statement on whether measurements were taken from distinct samples or whether the same sample was measured repeatedly
☐	☒	The statistical test(s) used AND whether they are one- or two-sided<br>*Only common tests should be described solely by name; describe more complex techniques in the Methods section.*
☐	☒	A description of all covariates tested
☐	☒	A description of any assumptions or corrections, such as tests of normality and adjustment for multiple comparisons
☐	☒	A full description of the statistical parameters including central tendency (e.g. means) or other basic estimates (e.g. regression coefficient) AND variation (e.g. standard deviation) or associated estimates of uncertainty (e.g. confidence intervals)
☐	☒	For null hypothesis testing, the test statistic (e.g. F, t, r) with confidence intervals, effect sizes, degrees of freedom and P value noted<br>*Give P values as exact values whenever suitable.*
☒	☐	For Bayesian analysis, information on the choice of priors and Markov chain Monte Carlo settings
☒	☐	For hierarchical and complex designs, identification of the appropriate level for tests and full reporting of outcomes
☒	☐	Estimates of effect sizes (e.g. Cohen's d, Pearson's r), indicating how they were calculated

Our web collection on statistics for biologists contains articles on many of the points above.

Software and code

Policy information about availability of computer code

Data collection	All the imaging data was collected through Leica TCS SP5 confocal and Leica SP8 Dive multiphoton system microscopes working on Leica LAS X software (version 3.7.4.23463). DiLiCre2.0 expression in tissue sections was analyzed using a Zeiss AxioScanner. WesternBlot was acquired from Fusion FX Spectra (Vilber).
Data analysis	All the imaging data in this article was analyzed with Fiji (National Institute of Health, version 1.53k) and Fiji scripting. Data processing is well indicated in Figure captions and Material and Methods. Additional algorithms used for data processing like the pixel classification through Weka segmentation (version 3.3.1) are provide as Supplementary Software. The parametric and non-parametric statistical analysis were performed in SPSS. The statistical comparisons of the curves evaluating the recombinant ratios over time were performed in RStudio (version 1.4.1103) using a permutation test based on the F-score from an Anova test, where we compare the original score to those obtained by permutation, using an empirical cumulative distribution to get the P values. All the data graphs included in this article were processed in GraphPad Prism version 9. Flow cytometry analysis was done in FlowJo (v10.6.1).

For manuscripts utilizing custom algorithms or software that are central to the research but not yet described in published literature, software must be made available to editors and reviewers. We strongly encourage code deposition in a community repository (e.g. GitHub). See the Nature Portfolio guidelines for submitting code & software for further information.

March 2021

Data

Policy information about availability of data

All manuscripts must include a data availability statement. This statement should provide the following information, where applicable:

- Accession codes, unique identifiers, or web links for publicly available datasets
- A description of any restrictions on data availability
- For clinical datasets or third party data, please ensure that the statement adheres to our policy

All manuscript data and image datasets were deposited at the The Netherlands Cancer Institute database

Field-specific reporting

Please select the one below that is the best fit for your research. If you are not sure, read the appropriate sections before making your selection.

[x] Life sciences [ ] Behavioural & social sciences [ ] Ecological, evolutionary & environmental sciences

For a reference copy of the document with all sections, see nature.com/documents/nr-reporting-summary-flat.pdf

Life sciences study design

All studies must disclose on these points even when the disclosure is negative.

Sample size	No sample-size calculation was performed due to the nature of the study.
Data exclusions	Only one data point was excluded in Extended Data Figure 3b. This data point shown abnormal high number of dead cells when analyzed by flow cytometry.
Replication	All the main experiments in this article were repeated at least 3 times independently. In addition, results were validated using orthogonal methods such as time-lapse confocal microscopy and flow cytometry in parallel. All the in-vivo experiments were performed including internal and external control conditions.
Randomization	Randomization was applied when selecting the positions to be exposed to the 405nm laser light. Other than that, no randomization was applied due to the nature of this study.
Blinding	Blinding was applied when collecting the organs for testing the expression of DiLiCre2.0 in the murine tissues. Other than that, no blinding was applied due to the nature of this study.

Reporting for specific materials, systems and methods

We require information from authors about some types of materials, experimental systems and methods used in many studies. Here, indicate whether each material, system or method listed is relevant to your study. If you are not sure if a list item applies to your research, read the appropriate section before selecting a response.

Materials & experimental systems

n/a	Involved in the study	
[ ]	[x]	Antibodies
[ ]	[x]	Eukaryotic cell lines
[x]	[ ]	Palaeontology and archaeology
[ ]	[x]	Animals and other organisms
[x]	[ ]	Human research participants
[x]	[ ]	Clinical data
[x]	[ ]	Dual use research of concern

Methods

n/a	Involved in the study	
[x]	[ ]	ChIP-seq
[ ]	[x]	Flow cytometry
[x]	[ ]	MRI-based neuroimaging

Antibodies

Antibodies used

Primary antibodies:
Target | Clone | Product number | Supplier | Host | Dilution
Cre | 2D8 | MAB3120 | Millipore | Mouse | 1:500
H3 | 1B1B2 | 14269 | CellSignaling | Mouse | 1:2000
HSP90 | C45G5 | 4877 | CellSignaling | Rabbit | 1:1000

Secondary antibodies:

	Target | Conjugate | Product number | Supplier | Dilution<br>Rabbit IgG | HRP | 32460 | ThermoFisher | 1:3000<br>Mouse IgG | HRP | 31430 | ThermoFisher | 1:3000
Validation	Validation of antibodies was done in HEK293T cell samples

Eukaryotic cell lines

Policy information about cell lines

Cell line source(s)	ATCC
Authentication	All cell lines were purchased from ATCC and none authentication was done.
Mycoplasma contamination	Regularly mycoplasma tests were performed in HEK293T and C26 cell lines. All resulted negative.
Commonly misidentified lines<br>(See ICLAC register)	Non commonly misidentified lines were used in this study.

Animals and other organisms

Policy information about studies involving animals; ARRIVE guidelines recommended for reporting animal research

Laboratory animals	Mus musculus B6 C57BL/6J females between 8-12 weeks old were used in this study.
Wild animals	No wild animals were used in this study.
Field-collected samples	No field-collected samples were used in this study.
Ethics oversight	All animals and experiments were conducted under the guidelines by the Animal Ethics Committee of the Netherlands Cancer Institute and performed in accordance with institutional, national and European guidelines for Animal Care and Use. All the animal protocols and surgery and imaging procedures were reviewed and approved by the Animal Care Committee of the Netherlands Cancer Institute (codes: 9.2.9867, 9.2.9917).

Note that full information on the approval of the study protocol must also be provided in the manuscript.

Flow Cytometry

Plots

Confirm that:

- [x] The axis labels state the marker and fluorochrome used (e.g. CD4-FITC).
- [x] The axis scales are clearly visible. Include numbers along axes only for bottom left plot of group (a 'group' is an analysis of identical markers).
- [x] All plots are contour plots with outliers or pseudocolor plots.
- [x] A numerical value for number of cells or percentage (with statistics) is provided.

Methodology

Sample preparation	Five to six days post doxycycline treatment, cells were resuspended, dissociated (TripLe, Thermo Fisher Scientific, cat. no. 12604013) filtered using test tubes with cell strainer snap cap (Thermo Fisher Scientific, cat. no. 352235) and FACS buffer (2% FBS and 2mM EDTA in PBS), and analyzed by FACS (LSRFortessa TM (BD Biosciences).
Instrument	FACS (LSRFortessa TM (BD Biosciences)
Software	FlowJo (v10.6.1)
Cell population abundance	ell population abundance and specificity was confirmed when analyzing Confetti cells by FACS. The different confetti cell populations were sorted using Aria Fusion instrument and subsequently validated by confocal microscopy.
Gating strategy	Gating strategy of Confetti flow cytometry analysis is provided in Figure 3.

- [x] Tick this box to confirm that a figure exemplifying the gating strategy is provided in the Supplementary Information.
