# Supplementary material for: A doxycycline- and light-inducible Cre recombinase mouse model for optogenetic genome editing
Source: Nat Commun. 2022 Oct 28;13:6442. doi: 10.1038/s41467-022-33863-z (PMC9616875; doi:10.1038/s41467-022-33863-z)
Supplement: Supplementary file 4 — Description of Additional Supplementary Files [file 41467_2022_33863_MOESM4_ESM.pdf]

Title: Supplementary Data 1.
Description: Summary table of HRASV12 effect on skin cell behaviour upon photoactivation (related to Fig. 6a-c and Supplementary Fig. 11a-b)

Title: Supplementary Movie 1.

Description: Time-lapse confocal movie of HEK293T cells that have been photoactivated in Figure 1 (panel c and d). A black boxed area denotes the photoconverted area.

Title: Supplementary Movie 2.
Description: Time-lapse confocal movie of C26 cells that have been photoactivated in Figure 1 (panel c and d). A black boxed area denotes the photoconverted area.

Title: Supplementary Movie 3.
Description: Time-lapse confocal movie of HEK293T cells that have been treated with doxycycline and non-exposed to violet-light (related to Supplementary Fig. 3e).

Title: Supplementary Movie 4.
Description: Time-lapse two-photon movie of the mouse ear skin upon DiLiCre2.0 photoactivation (related to Figure 5c and d). Within each imaging session, the photoactivated areas are annotated. Color code: fibrillar collagen I (magenta) and DiLiCre2.0 or HrasV12 (green).

Title: Supplementary Movie 5.
Description: Time-lapse two-photon movie of the mouse ear skin upon doxycycline treatment (related to Supplementary Fig. 10). Within each imaging session, the fluorescence intensity was measured within the same five randomly selected areas. Color code: fibrillar collagen I (magenta) and DiLiCre2.0 or HrasV12 (green).

Title: Supplementary Software

Description: ImageJ Weka Segmentation files. ImageJ Weka Segmentation (.arff and .model) and readme files are provided to know how to perform the segmentation of recombinant cells in timelapse confocal experiments.
